# Severe Metformin Intoxication Resulting in Extreme Lactic Acidosis

**DOI:** 10.14740/jmc5370

**Published:** 2026-09-04

**Authors:** Miriam Kozárová, Zuzana Kozelová, Slavomír Perečinský, Darko Švec, Lenka Šalamonová Blichová

**Affiliations:** a Fourth Internal Department, Medical Faculty, Pavol Jozef Šafárik University and Louis Pasteur University Hospital, Košice, Slovakia; b Department of Occupational Medicine and Clinical Toxicology, Medical Faculty, Pavol Jozef Šafárik University and Louis Pasteur University Hospital, Košice, Slovakia; c Medical Faculty, Pavol Jozef Šafárik University, Košice, Slovakia; d Department of Pathological Physiology, Medical Faculty, Pavol Jozef Šafárik University, Košice, Slovakia

**Keywords:** Metformin, Metformin-associated lactic acidosis, Intoxication, Hemodialysis, Acute kidney injury

## Abstract

The aim of this report is to present a case of massive intentional metformin overdose resulting in extreme metformin-associated lactic acidosis (MALA), acute kidney injury, recurrent cardiac arrest requiring cardiopulmonary resuscitation (CPR), and successful recovery including complete resolution of the transient sensorineural hearing loss following aggressive multidisciplinary management with extracorporeal elimination therapy. A 39-year-old woman intentionally ingested approximately 34 g of metformin (40 tablets of 850 mg) following an acute psychosocial conflict. She rapidly developed profound high-anion-gap metabolic acidosis with a nadir arterial pH of 6.86, peak serum lactate concentration of 41.7 mmol/L, progressive acute kidney injury with anuria, severe hemodynamic instability, and recurrent cardiac arrest requiring repeated CPR. Initial treatment with sodium bicarbonate was insufficient. Intensive care management included mechanical ventilation, intermittent hemodialysis, and continuous veno-venous hemofiltration (CVVH), resulting in gradual correction of metabolic abnormalities, recovery of renal function, resulting in complete clinical stabilization, including restoration of the transient sensorineural hearing loss. In conclusion, massive metformin overdose may lead to life-threatening lactic acidosis, multi-organ dysfunction, and circulatory collapse. This case illustrates that even patients presenting with extreme acidemia and hyperlactatemia can survive when severe MALA is recognized promptly and treated with early extracorporeal elimination combined with comprehensive intensive care.

## Introduction

Metformin remains the most widely prescribed first-line pharmacological treatment for type 2 diabetes mellitus because of its efficacy, favorable safety profile, low cost, and proven cardiovascular benefits. It primarily lowers blood glucose by suppressing hepatic gluconeogenesis and improving peripheral insulin sensitivity. Owing to its widespread use, more than 100–150 millions of patients worldwide are exposed to metformin, making awareness of its rare but potentially fatal adverse effects clinically important. Given its high household availability, metformin is readily accessible in both accidental and intentional overdose [1, 2].

Metformin is a hydrophilic cation with negligible plasma protein binding and an elimination half-life of approximately 5 h in individuals with preserved renal function. Metformin is eliminated largely unchanged by the kidneys through active tubular transport. Its disposition depends on organic cation transporters, particularly OCT1 in hepatocytes and OCT2 in renal tubular cells, together with multidrug and toxic extrusion (MATE) transporters responsible for tubular secretion into urine [2].

Metformin-associated lactic acidosis (MALA) is an uncommon but life-threatening complication characterized by high-anion-gap metabolic acidosis and elevated lactate in a patient exposed to metformin. Reported mortality remains high, particularly in critically ill patients and in those with organ failure or delayed initiation of extracorporeal treatment. Although epidemiological estimates suggest only 3–10 cases per 100,000 patient-years, the diagnosis is probably underrecognized because symptoms are non-specific and plasma metformin measurement is not routinely available [3–7].

The risk of MALA is highest when metformin exposure co-exists with factors that either increase lactate production or impair its clearance. The most important predisposing conditions include chronic renal impairment, acute kidney injury, severe infection or sepsis, tissue hypoperfusion, hepatic dysfunction, hypoxia, dehydration, excessive alcohol intake, and intentional overdose [3–7]. Acute poisoning may cause severe lactic acidosis even in previously healthy individuals without chronic kidney disease [6]. Risk factors of MALA are included in Table 1.

**Table 1:** Risk Factors of MALA

Risk factor	Comment
Renal impairment	Metformin is contraindicated when eGFR < 30 mL/min/1.73 m^2^. Renal function should be monitored regularly at eGFR 30–44 mL/min/1.73 m^2^; ≥ 45 mL/min/1.73 m^2^ is generally considered safe.
Hypoxia or tissue hypoperfusion	Heart failure, severe infection or sepsis, shock, and respiratory failure.
Hepatic disease	Liver dysfunction reduces lactate clearance.
Alcohol abuse	Alcohol increases lactate production and impairs gluconeogenesis.
Acute kidney injury	Often occurs with dehydration, vomiting, diarrhea, or sepsis.
Poisoning	Typically involves intentional overdose or suicide attempt, often in patients with renal impairment.

eGFR: estimated glomerular filtration rate; MALA: metformin-associated lactic acidosis.

Current pathophysiological models suggest that metformin disrupts lactate homeostasis through impaired hepatic gluconeogenesis and mitochondrial dysfunction. Experimental evidence indicates that inhibition of mitochondrial glycerol-3-phosphate dehydrogenase may alter the cytosolic redox state, reduce lactate-to-pyruvate conversion, and thereby diminish lactate utilization. In severe intoxication, this disturbance is amplified by renal failure, circulatory failure, and tissue hypoxia, producing profound acid-base balance disturbance [3–7].

The aim of this article is to present a case of intentional massive metformin poisoning complicated by extreme lactic acidosis, acute renal failure, and hemodynamic instability, and to discuss the pathogenesis, diagnostic approach, clinical presentation that includes also additional, rarely reported manifestation and therapeutic principles of severe MALA.

## Case Report

This report describes a single-patient case study of intentional metformin poisoning managed at Louis Pasteur University Hospital in Košice, Slovakia. Clinical data, arterial blood gas values, biochemical parameters, and details of treatment were retrospectively summarized from the medical record. The descriptive review of the literature was used only to contextualize the pathophysiology, diagnosis, and treatment of MALA. No experimental intervention was performed.

### Results

A 39-year-old woman with a history of psychiatric illness and previous suicidal behavior was admitted to the Emergency Department of Louis Pasteur University Hospital, Košice, Slovakia, on February 28, 2019 at 4 pm after intentionally ingesting approximately 34 g of metformin (40 tablets of 850 mg immediate-release metformin) following an acute psychosocial conflict in suicidal attempt. Intentional metformin overdose is a recognized cause of severe MALA, particularly after ingestion of large doses exceeding therapeutic exposure. Serum metformin concentration could not be determined because metformin-level testing was not available at our institution. As part of the differential diagnosis, alcohol intoxication and other potential causes of severe lactic acidosis, including sepsis, diabetic ketoacidosis, toxic alcohol ingestion, severe hypoxia, and hepatic failure, were considered and excluded.

On admission at 6:35 pm, the patient was conscious but rapidly developed nausea, vomiting, progressive weakness, tachypnea, and signs of circulatory instability. As part of the initial gastro-intestinal decontamination, activated charcoal was administered orally.

Initial arterial blood gas analysis demonstrated metabolic acidosis, which deteriorated rapidly during the following hours; sodium bicarbonate therapy was initiated, together with intravenous crystalloid administration.

**Figure 1:**
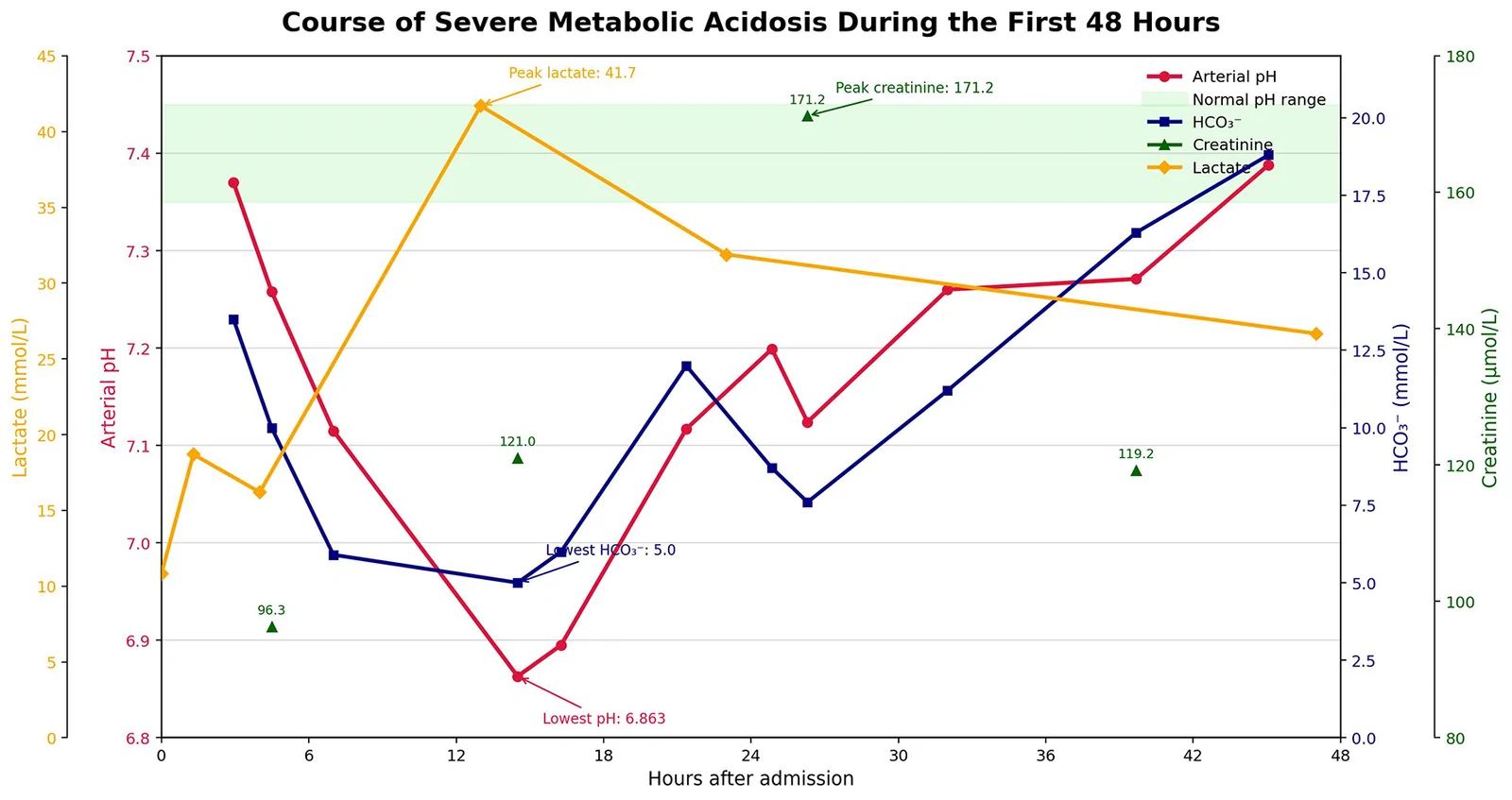
Course of severe metabolic acidosis during the first 48 h.

Within several hours after admission, the patient developed profound high-anion-gap metabolic acidosis with a nadir arterial pH of 6.86, bicarbonate concentration of 5.2 mmol/L, and base excess of −21 mmol/L. Peak serum lactate reached 41.7 mmol/L, representing one of the most severe biochemical manifestations of MALA. Laboratory findings within the first 48 h after admission are summarized in Figure 1.

Repetitive aboratory investigations demonstrated the progressive worsening of severe lactic metabolic acidosis. Therefore, urgent intermittent hemodialysis was indicated and performed using central venous catheter Certofix protect Trio HF V 1220.

Following completion of the first hemodialysis, the patient became psychomotorically agitated. After returning to the Intensive Care Unit of the Fourth Department of Internal Medicine, she was uncooperative and unable to follow instructions, producing only non-articulated sounds. Recurrent episodes of hypoglycemia were corrected by continuous intravenous glucose administration that led to temporary improvement of the consciousness. She subsequently complained of thirst and fatigue, while mild psychomotor agitation persisted.

On March 1, 2019 at 07:00 am (12 h after admission), the patient gradually developed sinus bradycardia, arterial hypotension and recurrent deterioration of consciousness, with agonal respirations. Follow-up laboratory investigations confirmed further progression of the metabolic acidosis despite the preceding hemodialysis treatment. She was ventilated using a bag-valve mask, and atropine 1 mg intravenously and adrenaline 1 mg were administered. A brief episode of ventricular tachycardia occurred on bed-side monitor. Following approximately 5 min of cardiopulmonary resuscitation (CPR), spontaneous cardiac rhythm was restored. The patient was intubated and required catecholamine support with noradrenalin intravenously. She was subsequently transferred to the Department of Anesthesiology and Intensive Care Medicine.

On admission to the Department of Anesthesiology and Intensive Care Medicine on March 1, 2019 at 8:00 am, the patient was in profound shock, with severe hemodynamic instability requiring dual vasopressor support with noradrenaline and vasopressin at high rates. She was analgosedated, mechanically ventilated and had extreme metabolic lactic acidosis. Continuous veno-venous hemodialysis (CVVH) was initiated, together with aggressive intravenous fluid therapy. Treatment parameters of CVVH were blood flow rate of 150 mL/min; dialysate flow rate of 3,000 mL/h, and a potassium concentration of 4.0 mmol/L with minimal ultrafiltration.

Anticoagulation with unfractionated heparin, 5,000 IU as an initial bolus, was followed by continuous infusion at 1,000 IU/h, with APTT monitoring and a target of 1.5–2 times the normal value. Spontaneous urine output was initially preserved.

A gradual stabilization of the circulation and ventilatory parameters was achieved. The patient was successfully extubated on March 3, 2019. During the first few days, co-operation was markedly limited due to severe psychomotor agitation, requiring repeated sedation with tiapride.

After resolution of the delirious state, the patient reported transient auditory distortion described as echo-like hearing. An otorhinolaryngology consultation was performed, with the conclusion that an organic hearing disorder was unlikely. The hearing impairment gradually resolved without specific treatment within the next 20 days.

Due to elevated inflammatory markers and leukocytosis with neutrophilia, amoxicillin/clavulanic acid was added to the treatment. After 72 h, CVVH was discontinued as planned following marked improvement in acid-base status. Vasopressor support was discontinued as well. The course of severe metabolic acidosis during the first 48 h is shown in Figure 1.

Chest radiography demonstrated a significant right-sided pleural effusion, which was also confirmed by ultrasonography. After improvement in the platelet count, a right-sided pleural drain was inserted using a Pleuracan catheter, and approximately 1,200 mL of serous fluid was evacuated. Follow-up chest radiography also demonstrated a left-sided pleural effusion.

Initially, elevated liver enzyme levels gradually normalized. Oral nutritional intake could not be established because the patient reported marked loss of appetite and refused food. Supplementary parenteral nutrition was therefore continued.

**Figure 2:**
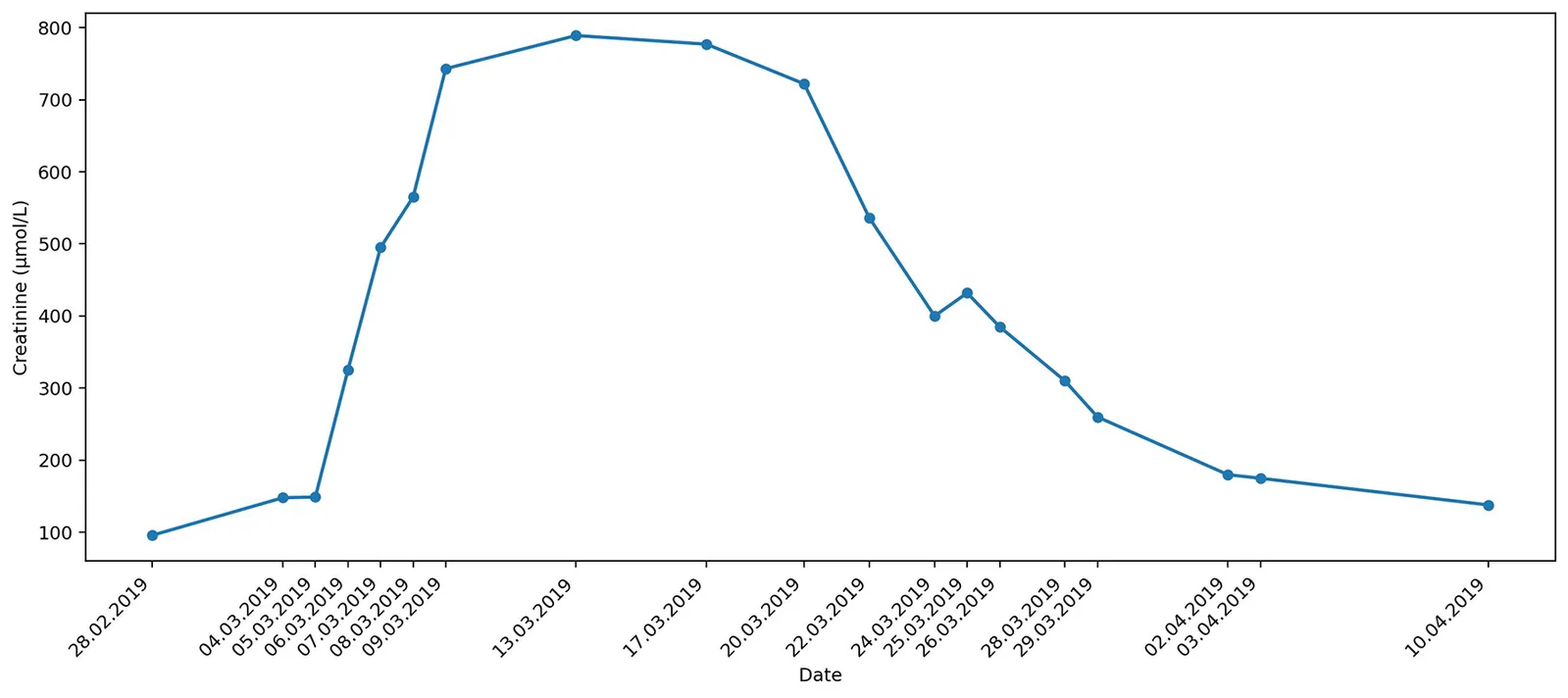
Serum creatinine levels during MALA. MALA: metformin-associated lactic acidosis.

Because of persistent anuria, intermittent hemodialysis was performed on March 7, 2019 and was well tolerated. Serial arterial blood gas analyses demonstrated during the subsequent days of hospital stay gradual normalization of pH, decreasing serum lactate concentrations, and progressive recovery of renal function. The course of serum creatinine levels during MALA is shown in Figure 2. Following stabilization of hemodynamics, renal replacement therapy was discontinued. After recovery, she was transferred for further psychiatric management to the Department of Psychiatry on March 29, 2019 with no permanent neurological deficit and with resolved sensorineural hearing disorder. The changes in laboratory parameters during hospitalization are shown chronologically in Table 2.

**Table 2:** Laboratory Parameters During Hospitalization

Time	Clinical event	pH	Serum lactate (mmol/L)	Serum creatinine (µmol/L)	Treatment
0 h	Ingestion of 34 g metformin	N/A	N/A	N/A	N/A
ED admission	Initial assessment	7.37	10.8	96.3	Fluids
+2 h	Progressive acidosis	7.26	18.7	121	Sodium bicarbonate
+12 h	Clinical deterioration	6.86	41.7	148.7	CPR
+14 h ICU admission	Recurrent cardiac arrest	6.89	39	171.2	Mechanical ventilation
14–24 h ICU	Intermittent hemodialysis	7.12	↓	171.2	Intermittent hemodialysis
Department of Anesthesiology days 2–3	CVVH	7.27	↓	119.2	CVVH
ICU days 7–29	Acute renal failure with anuria	7.39	< 3	Highest level 738	Intermittent hemodialysis
ICU day 29	Recovery	7.37	0.66	260	Discharge from ICU after 29 days

The clinical course was reconstructed chronologically from the available medical records. CPR: cardiopulmonary resuscitation; CVVH: continuous veno-venous hemofiltration; ED: emergency department; h: hours; ICU: intensive care unit; pH: arterial blood pH; N/A: not available; ↓: decrease.

## Discussion

MALA is a rare but potentially fatal toxicological emergency [1–3, 5]. Its reported incidence is approximately 3–10 cases per 100,000 patient-years, but mortality remains substantial, particularly in patients presenting with circulatory shock, acute kidney injury, severe acidemia, or delayed initiation of extracorporeal treatment [1, 3, 5, 8, 9]. Given its widespread use and household availability, metformin remains accessible for both accidental ingestion and intentional overdose [5, 10–16].

The presented patient intentionally ingested approximately 34 g of metformin and subsequently developed profound metabolic acidosis, with a nadir arterial pH of 6.86 and a peak lactate concentration of 41.7 mmol/L. The clinical course was further complicated by anuric acute kidney injury, hemodynamic collapse, recurrent cardiac arrest requiring repeated CPR, mechanical ventilation, and renal replacement therapy. Although MALA has been extensively described, relatively few reports document survival with complete neurological recovery after the simultaneous occurrence of such extreme hyperlactatemia, profound acidemia, anuria, and recurrent cardiac arrest [7, 8, 10–17]. Survival despite extremely low arterial pH and severe hyperlactatemia has nevertheless been reported, including survival after a metformin overdose resulting in a serum pH of 6.59 [11]. This case therefore demonstrates that favorable outcomes remain possible even in patients with biochemical abnormalities that may initially appear incompatible with survival, provided that the diagnosis is recognized promptly and extracorporeal treatment is initiated without delay [9, 11, 17].

The pathophysiology of MALA is multifactorial. Metformin interferes with mitochondrial oxidative metabolism, including inhibition of respiratory chain complex I, resulting in reduced adenosine triphosphate production and increased reliance on anaerobic metabolic pathways [2, 3]. Additional mechanisms involve alterations in mitochondrial redox pathways, impairment of hepatic gluconeogenesis, and reduced hepatic lactate utilization, thereby promoting progressive lactate accumulation and severe high-anion-gap metabolic acidosis [2, 3]. In massive overdose, metformin-induced metabolic dysfunction may be further aggravated by tissue hypoperfusion, hemodynamic instability, and acute kidney injury [5, 7, 8, 14]. Because metformin is eliminated predominantly unchanged by the kidneys, impaired renal function substantially reduces its clearance and may establish a self-perpetuating cycle of drug accumulation, worsening acidosis, cardiovascular dysfunction, and multi-organ failure [2, 4, 5].

The diagnosis of MALA is primarily clinical and should be considered in any patient with known or suspected metformin exposure who develops otherwise unexplained high-anion-gap metabolic acidosis and an elevated lactate concentration, typically above 5 mmol/L [1, 5, 9]. Measurement of plasma metformin concentration may support the diagnosis; however, such testing is frequently unavailable in emergency settings, results may not be available sufficiently rapidly to guide acute management, and the correlation between metformin concentration and clinical severity is imperfect [5, 9, 15]. Dell’Aglio et al demonstrated considerable overlap in serum metformin concentrations, lactate levels, and pH values between survivors and non-survivors of acute metformin overdose, underscoring the limitations of relying on an isolated metformin concentration for prognostication [15]. In the present case, serum metformin concentration could not be determined because metformin level testing was not available at our institution, which we consider the major limitation of the reported case. The diagnosis was therefore based on the documented history of massive metformin ingestion, the characteristic biochemical findings, rapidly progressive renal dysfunction, and the exclusion of other plausible causes of profound lactic acidosis.

The differential diagnosis included septic shock, diabetic ketoacidosis, toxic alcohol ingestion, hepatic failure, and severe tissue hypoxia. However, these conditions were not supported by the clinical history, laboratory findings, or overall temporal course. The early gastro-intestinal symptoms, rapidly progressive acidemia, marked hyperlactatemia, and evolving anuric renal failure following a confirmed massive metformin ingestion were strongly consistent with severe acute metformin toxicity [5, 7, 8, 12–14].

The principal therapeutic consideration in this case was the timing of extracorporeal treatment. Initial supportive measures, including intravenous fluid administration, hemodynamic support, mechanical ventilation, correction of hypoglycemia, and sodium bicarbonate administration, were insufficient to reverse the rapidly progressive metabolic deterioration. Although bicarbonate may provide temporary buffering in selected patients with life-threatening acidemia, it does not remove metformin and should not delay definitive extracorporeal therapy [5, 9].

According to the Extracorporeal Treatments in Poisoning (EXTRIP) Workgroup recommendations, extracorporeal treatment is indicated in severe metformin poisoning when the lactate concentration exceeds 20 mmol/L, arterial pH is ≤ 7.0, standard supportive treatment fails, or severe clinical manifestations such as shock, renal impairment, decreased consciousness, or liver failure are present [9]. Extracorporeal treatment is also suggested at lactate concentrations of 15–20 mmol/L or arterial pH values of 7.0–7.1 when additional factors associated with poor prognosis are present [9]. Our patient fulfilled several major criteria for immediate extracorporeal treatment, including a lactate concentration exceeding 40 mmol/L, arterial pH below 6.9, anuric acute kidney injury, refractory hemodynamic instability, and recurrent cardiac arrest.

Intermittent hemodialysis is generally considered the preferred initial extracorporeal modality in severe MALA because it provides rapid metformin clearance and efficient correction of acid-base and electrolyte disturbances [9]. In the present case, intermittent hemodialysis was followed by CVVH because persistent hemodynamic instability required ongoing extracorporeal support. A sequential or prolonged extracorporeal approach may be necessary in critically ill patients because metformin has a large volume of distribution and redistribution from peripheral compartments may produce rebound toxicity after initial dialysis [2, 9, 17]. High-flow or high-volume hemodiafiltration has also been successfully used in patients with severe MALA and circulatory failure [17]. This sequential approach permitted rapid initial correction while maintaining continuous control of acidosis and metabolic abnormalities during the subsequent critical period. Extracorporeal treatment may generally be discontinued when the lactate concentration has decreased below 3 mmol/L and arterial pH has increased above 7.35, although close monitoring is required because redistribution of metformin from peripheral tissues may result in rebound acidosis [9].

Observational evidence and accumulated case experience indicate that patients receiving extracorporeal treatment often present with more severe acidemia, higher lactate concentrations, hemodynamic instability, and greater organ dysfunction than patients managed conservatively [5, 9, 17]. Despite this greater baseline severity, favorable outcomes have repeatedly been described when extracorporeal treatment is instituted promptly [7–9, 11, 14, 17]. Conversely, selected patients with less severe acute overdose may recover with conservative treatment alone, as demonstrated in individual case reports [10]. These findings support an early dialysis-based strategy in severe MALA rather than prolonged conservative management in patients with progressive metabolic deterioration, while recognizing that treatment should be individualized according to clinical severity [9].

The ingested dose of approximately 34 g was massive, although it was not the largest acute metformin overdose reported in the literature [11–13, 15, 16]. The clinical contribution of this report therefore does not depend solely on the quantity ingested. Previous reports have shown that the relationship between ingested dose, serum metformin concentration, lactate concentration, arterial pH, and outcome is complex, and no single biochemical variable reliably determines prognosis [12, 15].

The patient also developed a transient auditory disturbance described as echo-like hearing. A direct causal relationship between metformin and this symptom cannot be established. Auditory dysfunction following severe poisoning may be multifactorial and could reflect profound hypotension, cochlear hypoperfusion, metabolic disturbance, hypoxia, or the effects of concomitant treatment. Miller et al described persistent bilateral sensorineural hearing loss following an intentional combined overdose of 52 g of metformin and 350 mg of glyburide associated with severe lactic acidosis, hypotension, respiratory failure, and acute renal failure [16]. In that report, severe hypotension and consequent cochlear hypoperfusion were considered plausible contributors to auditory injury [16]. In contrast, the auditory symptoms in our patient resolved spontaneously, suggesting a transient functional disturbance rather than permanent cochlear injury.

Nevertheless, this observation should be interpreted cautiously because formal audiological testing was not performed during the acute phase. The relationship between metformin and auditory function is particularly difficult to interpret because the available evidence is heterogeneous. While sensorineural hearing loss has been reported after severe intentional metformin overdose complicated by hypotension and multi-organ failure [16], a large observational study found that metformin use in patients with diabetes mellitus was associated with a lower risk of sudden sensorineural hearing loss [18]. Accordingly, the transient auditory disturbance observed in our patient should not be interpreted as evidence of a direct ototoxic effect of metformin.

In conclusion, this case demonstrates survival after an exceptionally severe acute metformin overdose characterized by profound acidemia, extreme hyperlactatemia, anuric renal failure, recurrent cardiac arrest, and multi-organ dysfunction. Early recognition, rapid laboratory assessment, aggressive supportive intensive care, and timely extracorporeal elimination were central to the favorable outcome [5, 9, 17]. The case reinforces the practical message that even extreme biochemical abnormalities should not automatically be regarded as futile, as survival without major neurological sequelae has been documented even after extraordinarily severe acidemia [11, 15]. Prompt multidisciplinary treatment may therefore be lifesaving in patients with severe MALA. The clinical presentation was also notable for a transient auditory disturbance, a rarely reported manifestation in the setting of severe metformin intoxication [16].

### Conclusions

Massive metformin overdose may rapidly lead to extreme lactic acidosis, acute kidney injury, and life-threatening hemodynamic instability. In patients with severe MALA, rapid diagnosis, intensive supportive care, and timely extracorporeal elimination are essential. The presented case underlines the decisive role of hemodialysis and continuous renal replacement therapy when sodium bicarbonate and standard resuscitative measures are insufficient.

## Data Availability

The authors declare that data supporting the findings of this case report are available within the article.
